# Remnant Cholesterol as an Independent Predictor of Periodontitis: A Population-Based Study

**DOI:** 10.1155/2023/3413356

**Published:** 2023-02-14

**Authors:** Qian Peng, Yiwen Xiao, Zhiguo Tian, Yunwei Yang, Jiang Deng, Jie Lin

**Affiliations:** ^1^Department of Plastic and Reconstructive Surgery, Hubei NO. 3 People's Hospital of Jianghan University, Wuhan 430000, China; ^2^Department of Stomatology, Hubei NO. 3 People's Hospital of Jianghan University, Wuhan 430000, China; ^3^Department of Urology, Hubei NO. 3 People's Hospital of Jianghan University, Wuhan 430000, China; ^4^Department of Pharmacy, Hubei NO. 3 People's Hospital of Jianghan University, Wuhan 430000, China

## Abstract

**Objective:**

Numerus studies present that remnant cholesterol (RC) as a risk factor participates in the progression of multiple diseases. The aim of this study was to assess the relationship between cholesterol and periodontitis in the US population to find a reliable lipid predictor for periodontitis.

**Materials and Methods:**

Clinical data was retrieved from the National Health and Nutrition Examination Survey (NHANES) database between 2009 and 2014. The logistic regression was conducted to examine the corelationship between RC and various clinical features. Meanwhile, the dose-response relationship was measured through restricted cubic spline analysis. And the propensity score matching (PSM) was established to further investigate the potential relationship between RC and periodontitis.

**Results:**

A number of 4,829 eligible participants were included in this study. It was found that the increased RC is associated with the higher risk of periodontitis after adjusting the potential confounding factors with the adjusted odds ratios (aOR) of 1.403 (95% confidence intervals (CI): 1.171-1.681, *P* < 0.001, univariate analysis) and 1.341 (95% CI: 1.105-1.629, *P* = 0.003, multivariate analysis) in the highest grade. There were significant differences in the relationship between RC and various clinical features including age, gender, body mass index (BMI), race, hypertension, and diabetes mellitus (all *P* < 0.001). Besides, the calculated thresholds for predicting periodontitis were 19.99 (before propensity score matching (PSM)) and 20.91 (after PSM) mg/dL.

**Conclusions:**

In this study, RC was identified to be positively associated with the occurrence of periodontitis, which suggests that RC can be considered as a predictor for periodontitis.

## 1. Introduction

Periodontitis is one of the most ubiquitous oral diseases, with a high prevalence of up to 90% worldwide [1]. Periodontitis is a chronic inflammatory disease initiated by periodontal bacterial infection [2], and its symptoms include red, bleeding, or swollen gums. Periodontitis afflicts the periodontium, leads to alveolar bone resorption, and eventually results in tooth mobility [1]. Additionally, gingival recessions and tooth loss further cause impaired esthetics [3]. Patients with periodontitis always suffer substantial functional, physical, psychological, and social impacts [4]. Therefore, it is urgent to identify an ideal and appropriate diagnostic biomarker for periodontitis as early as practicable.

During the past few years, great efforts have been made to explore useful potential biological indices that can predict the presence of periodontitis before extensive periodontal damage has occurred [5]. Since the advantage of noninvasively accessible, biomarker from saliva has been demonstrated to be used for early detection [6, 7]. For example, there were significant difference of gingival crevicular fluid between the periodontitis group and the control group [8].

However, considering the difficulty in probing the pocket depths of teeth for community screening, developing a convenient and effective periodontitis biomarker would be more desirable [5]. In a study on the early diagnosis of oral diseases, Currò et al. found that transglutaminase gene expression may be modified in response to chronic injury to damaged gingiva and highlighted the critical role of these enzymes in gingival repair, healing, and adaptation [9]. In addition, metagenomics and 16S gene analysis methods to analyze group differences in the periodontal microbiome become useful to contribute to the early diagnosis of periodontitis [10]. Isola et al. revealed that periodontitis was negatively associated with low serum endothelial progenitor cell (CD133^+^/KDR^+^) levels. Periodontitis may have negatively affected CD133^+^/KDR^+^ levels [11]. Previous studies have showed that intensive periodontal therapy ameliorates both local (periodontal) and systemic inflammation [12]. In other words, as a local oral inflammatory disease, periodontitis has a possible link between a number of systemic inflammatory diseases, such as cardiovascular diseases, atherosclerosis, and type 2 diabetes [12–14]. Currently, new insight suggests that remnant cholesterol (RC) is a strong and independent predictor of atherosclerotic cardiovascular disease [15, 16]. The term RC represents the cholesterol content of all triglyceride-rich lipoproteins, which refers to total cholesterol minus both low-density lipoprotein and high-density lipoprotein. Remnant particles circulate in plasma and accumulate in the subendothelial space, potentially propagating local and systemic inflammation, ultimately contributing to endothelial dysfunction and atherogenesis [15, 17].

RC plays a key role in the inflammatory and atherogenesis process. An observational study suggested the atherogenicity of RC with an important inflammatory component, contributing to the increased cardiovascular disease risk [17]. In addition, the Copenhagen General Population Study including 60608 individuals reported that elevated RC level caused both low-grade inflammation and ischemic heart disease [18]. Moreover, severe periodontal disease was revealed to be associated with increased aortic arch atheroma thickness and calcification in an epidemiological survey that included 106 patients [19]. However, few data described the association between RC level and periodontitis risk. In order to investigate more plausible and reliable lipid markers for periodontitis and to enhance our understanding of the pathophysiological mechanisms of periodontitis, we used data on US adults from the National Health and Nutrition Examination Survey (NHANES) conducted between 2009 and 2014 to investigate the relationship between RC level and periodontitis. And this study identifies the diagnostic value and current application of RC as a new indicator for the diagnosis and prediction of periodontitis.

## 2. Materials and Methods

### 2.1. Participants

The demographic data included in the study originates from National Health and Nutrition Examination Survey (NHANES). Study protocols of NHANES were approved by the Institutional Review Board of National Center for Health Statistics (NCHS), and participants' consent was obtained during the survey process.

**Figure 1:**
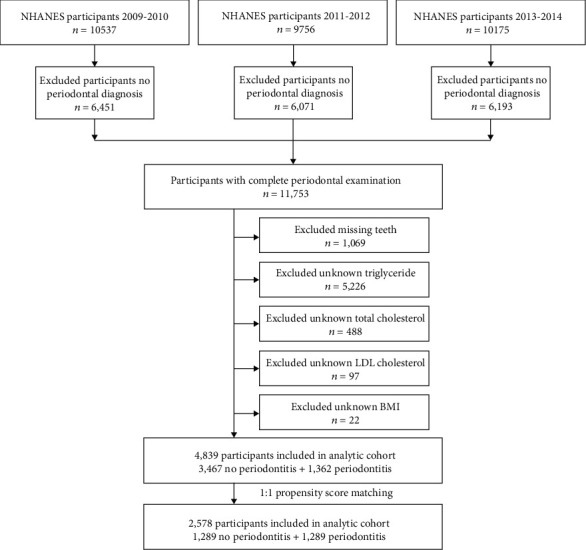
Schematic flow diagram introducing exclusion criteria in this study.

In this study, we analyzed data from three consecutive NHANES 2-year survey cycles (2009–2010, 2011–2012, and 2013–2014). There were 11,753 participants with complete periodontal examination from NHANES from 2009 to 2014. Considering the potential influencing factors, the exclusion criteria followed in this study were as follows: (1) with missing teeth; (2) unknown triglyceride; (3) unknown total cholesterol; (4) unknown low-density lipoprotein cholesterol (LDL-C); and (5) unknown body mass index (BMI) (Figure 1). A total of 4839 participants were subsequently included in the 1 : 1 propensity score matching analysis based on characteristics such as gender, marital, race, history of hypertension, and history of diabetes. A total of 2578 participants were eventually included in our study.

### 2.2. Clinical Variables

The clinical variables including age, gender, BMI, marital status, race, hypertension, diabetes mellitus, periodontitis, total cholesterol (TC), triglycerides (TG), high-density lipoprotein cholesterol (HDL-C), and LDL-C were obtained from NHANES. For the determination of periodontitis, the judgment criteria in our work were defined as “≥ two interproximal sites accompanied with attachment loss (AL) ≥3 mm, ≥ two interproximal sites accompanied with probing depth (PD) ≥4 mm not on the same tooth, or one site accompanied with PD ≥5 mm” [20]. And the detailed data of AL and PD were obtained from NHANES (https://www.cdc.gov/nchs/nhanes/index.htm). Based on the laboratory procedures manual, TC and TG were collected through the enzymatic assays, and HDL-C was measured through immunoassays. As for LDL-C, it was determined according to the Friedewald calculation. Meanwhile, the measurement of RC was calculated by TC minus LDL-C minus HDL-C [21].

### 2.3. Statistical Analysis

Continuous variables were represented by mean ± standard deviation, while the classified variables were counted by proportion in the study. Chi-square analysis was conducted to declare the characteristics of all qualified participants. Logistic regression was performed to determine the odds ratios (ORs) with 95% confidence interval (CI). Univariate and multivariate models were established for the evaluation of relationship between RC and periodontitis. Restricted cubic spline (RCS) was conducted to analyze the complex relationship between RC [22]. The occurrence of periodontitis is measured rigorously based on the instruction. 1 : 1 propensity score matching (PSM) was conducted to match age, gender, race, marital status, BMI, diabetes mellitus, and hypertension. The standardized difference of each variable was calculated and found to be less than 0.1. All the analyses were conducted by using R (version 3.5.3) and SPSS software (version 24.0) with two-tailed *P* < 0.05 considered as statistically significant.

## 3. Results

### 3.1. Demographic Characteristics

There were 4829 participants retrieved from NHANES 2007–2014 in the study. As shown in Table 1, the participants were divided into four groups according to the grade of RC. Chi-square analysis indicated that there were several significant differences among variables, including age, gender, BMI, race, hypertension, diabetes mellitus, periodontitis, total cholesterol, triglycerides, HDL-C, and LDL-C (all *P* value < 0.001). Participants with higher grade of RC tended to be older and male. And distributions of non-Hispanic white, hypertension, and diabetes mellitus were gradually increasing with the grade of RC. Intriguingly, with the elevation of RC grade, the prevalence of periodontitis decreased slightly. Moreover, participants with higher grade of RC had higher total cholesterol, triglycerides, LDL-C, and lower HDL-C.

### 3.2. Relationship between RC and Periodontitis

As shown in Table 2, logistical regression was performed to analyze the relationship between RC and the risk of periodontitis. Demographic characteristics were adjusted, and results showed that RC was the risk factor for periodontitis in univariate analysis (*P* = 0.003). The adjusted odds ratios (aOR) in higher levels of RC were 1.220 (95% CI: 1.019-1.461) and 1.403 (95% CI:1.171-1.681), respectively, compared to the lowest level of RC. Similarly, the multivariate analysis illustrated the positive associations between RC level and the occurrence of periodontitis (*P* = 0.027), and the aOR in the highest level of RC was 1.341 (95% CI: 1.105-1.629) compared with the lowest level of RC.

### 3.3. Restricted Cubic Spline to Determine the Optimal Cutoff Values of RC for Periodontitis

**Figure 2:**
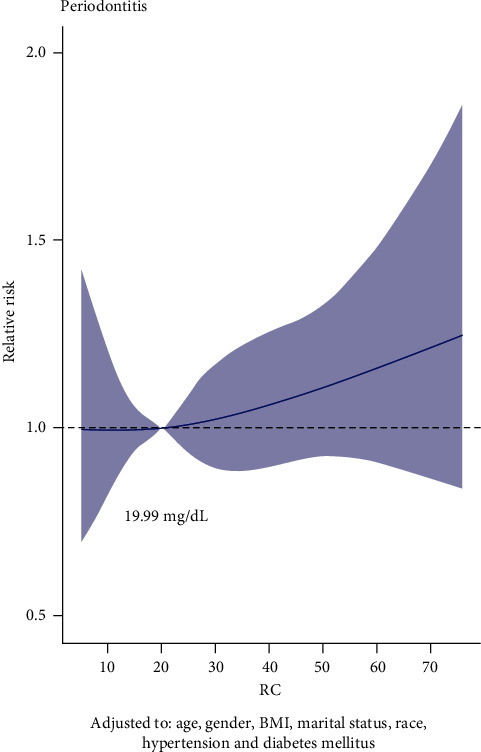
The dose-response analysis between RC and periodontitis. Adjusted to age, gender, BMI, material status, race, hypertension, and diabetes mellitus.

**Figure 3:**
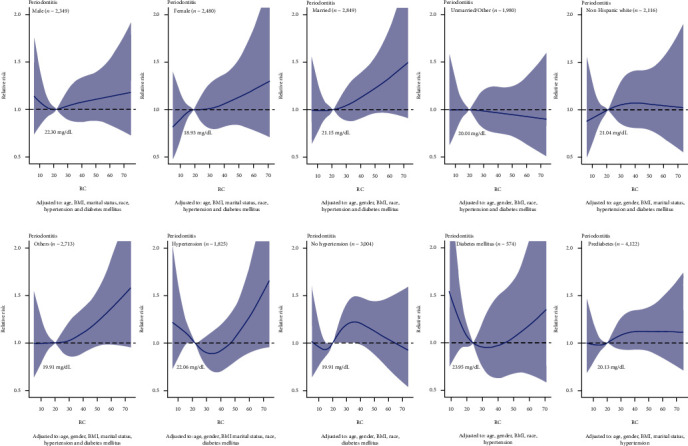
The dose-response analysis between RC and periodontitis among the different subgroups, including gender, material status, race, hypertension, and diabetes mellitus.

Figure 2 shows a significant nonlinear dose-response association between RC and periodontitis, adjusting age, gender, BMI, marital status, race, hypertension, and diabetes mellitus. The RCS model demonstrated the relative periodontitis risk corresponding to different levels of RC compared to 19.99 mg/dL. We also used subgroup analysis to simulate the relationship between RC and periodontitis, based on the stratification of these confounders. Similar positive relationships between RC and periodontitis were found in subgroups of females, the married, and races other than the non-Hispanic white (Figure 3).

**Figure 4:**
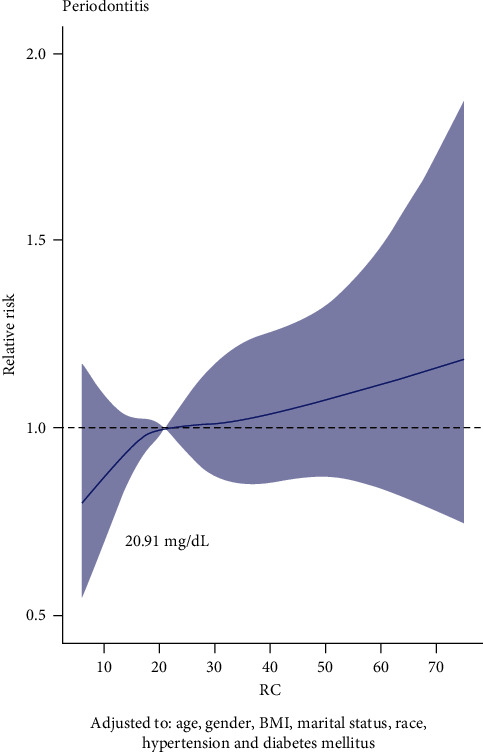
The dose-response analysis between RC and periodontitis after 1 : 1 propensity score matching. Adjusted to age, gender, BMI, material status, race, hypertension, and diabetes mellitus.

**Figure 5:**
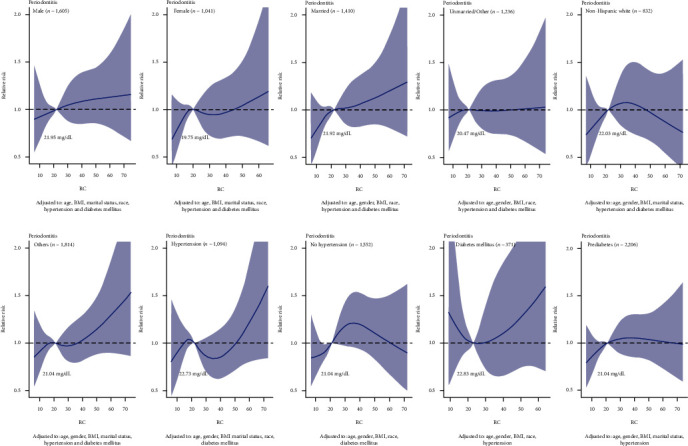
The dose-response analysis between RC and periodontitis among the different subgroups after 1 : 1 propensity score matching.

Furthermore, we screened the well-balanced pairs using the 1 : 1 PSM to eliminate standardized mean difference (Supplementary Figure 1). After PSM, the association between RC and periodontitis was still significantly positive compared to 20.91 mg/dL of RC (Figure 4). Intriguingly, positive relationship between RC and periodontitis was found in subgroups of males after PSM, which was opposite to the results before PSM. However, for those married patients, the correlation remained the same as before (Figure 5).

## 4. Discussion

Periodontitis exerts a negative impact on the quality of life by compromising aspects related to function and esthetics, as the disease has been related to eating difficulty, pain, tooth loss, and changes in facial appearance [23]. The potential mechanism might originate from the excess oxidative stress induced by the inflammation which damaged the normal function [24, 25]. Considering the severe damage to the dentition, it is important to identify and intercept periodontitis early. As a convenient and inexpensive biological factor, RC level has been proved to be positively related to cardiovascular diseases, diabetes, and so on, but its role in predicting periodontitis is still unclear. In this study, we analyze the relationship between RC and periodontitis based on NHANES database and found that the levels of RC were positively related with occurrence of periodontitis. Moreover, when adjusted demographic characteristics, logistical regression results indicated that participants with higher RC levels tended to have higher occurrences of periodontitis. And RCS suggested the optimal cutoff values of RC for periodontitis were 19.99 mg/dL and 20.91 mg/dL, respectively, before or after PSM. Therefore, RC could serve as a reliable biological factor to predict the occurrence of periodontitis.

Although this study demonstrated the positive relationship between RC and occurrence of periodontitis, the potential molecular mechanism remains unclear and needs to be explored. We assumed that inflammation mediated by RC plays a crucial role in the process. RC refers to the cholesterol content of triglyceride-rich lipoproteins, including very low-density and intermediate-density lipoproteins in the fasting state. In recent years, RC has emerged as an independent predictor in many diseases, such as ischemic heart disease, diabetes, and nonalcoholic fatty liver disease. However, the relationship between RC and periodontitis remains unknown. Varbo et al. found a bidirectional relationship between elevated RC and elevated CRP levels in ischemic heart disease, indicating a causal association between RC and inflammation [18]. Notably, several researches have declared the linkage between periodontitis and inflammation: Leira et al. demonstrated that severity of periodontitis was associated with elevated degree of hs-CRP and IL-6 in a case-control study of neurodegenerative diseases [26]; Gomes-Filho et al. found increasing level of CRP in participants with periodontitis, who may have higher risk of acute myocardial infarction [27]. These results emphasized the potential role of inflammation in the pathology of periodontitis. Overall, inflammation mediated by CRP/IL-6 may connect RC and periodontitis occurrence. However, NHANES database did not include CRP or IL-6 level, and the concrete mechanism needs verifying in cellular or animal experiments.

In this study, RCS analysis was performed to explore the association between RC and periodontitis. RCS analysis could flexibly model complex or nonlinear relationship between variables and outcomes. RCS analysis revealed that RC and the occurrence of periodontitis existed a nonlinear and positive association. Optimal cutoffs of RC in this study may be useful in predicting the occurrence of periodontitis. Additionally, PSM was performed to eliminate potential confounding variables. It is confused that RC was positively related to periodontitis in females before PSM, while RC was positively related to periodontitis in males after PSM. This may attribute to the reason that females were more than males before PSM, and after PSM, the significance of RC in males emerged.

Management of periodontitis requires a combination of therapeutic modalities, which includes subgingival instrumentation to remove plaque and calculus, various types of surgery, and local and systemic pharmacotherapy [23]. Previous research exhibited that drugs reducing RC and resulting in a favorable lipid/lipoprotein profile can decrease the risk of cardiovascular disease [28]. Based on our study that RC can be considered as the predictor for the occurrence of periodontitis, whether drugs to diminish RC can also be used as a pharmacotherapy for periodontitis needs further international and clinical verification. It may provide a potential strategy for direction in periodontitis.

However, there are some limitations in this study. First, as a retrospective research, the bias is inevitable even when PSM was performed. Second, the RC cutoff value for predicting target outcomes in patients with periodontitis may only be appropriate for the data in this study and may rarely be replicated in other independent data. Therefore, reproduction of the cutoff values is needed in future studies, and multicenter prospective studies are needed to validate this conclusion. Furthermore, the relationship between RC levels and severity of periodontitis could be explored to stratify the patients for the target outcomes.

## 5. Conclusions

This study evaluated the relationship between RC and periodontitis in the nationally representative cross-sectional NHANES database. After adjusting for potential confounding variables, it was found that RC was indeed positively correlated with the occurrence of periodontitis. The newly established cutoff values should be considered to predict periodontitis. Therefore, it is important to manage blood cholesterol to reduce the risk of periodontitis by focusing not only on traditional lipid parameters but also on RC levels.

## Figures and Tables

**Table 1:** Baseline characteristics of 4829 participants.

Characteristic	Total	RC (mg/dL)				*P* value
		Grade 1 (<15)	Grade 2 (15~20)	Grade 3 (20~30)	Grade 4 (>30)	
	No. (%)	No. (%)	No. (%)	No. (%)	No. (%)	
Total patients	4829	1185 (24.5)	1245 (25.8)	1243 (25.7)	1156 (23.9)	
Age (IQR)	40, 63	39, 61	40, 63	42, 65	41, 62	<0.001
Gender						<0.001
Male	2349 (48.6)	487 (41.1)	599 (48.1)	609 (49.0)	654 (56.6)	
Female	2480 (51.4)	698 (58.9)	646 (51.9)	634 (51.0)	502 (43.4)	
BMI (kg/m^2^) (IQR)	24.60, 32.41	22.80, 30.50	23.91, 31.58	25.60, 33.10	26.89, 34.20	<0.001
Marital status						0.694
Married	2849 (59.0)	706 (59.6)	717 (57.6)	742 (59.7)	684 (59.2)	
Unmarried/other	1980 (41.0)	479 (40.4)	528 (42.4)	501 (40.3)	472 (40.8)	
Race						<0.001
Non-Hispanic white	2116 (43.8)	495 (41.8)	542 (43.5)	536 (43.1)	543 (47.0)	
Non-Hispanic black	942 (19.5)	353 (29.8)	277 (22.2)	191 (15.4)	121 (18.2)	
Mexican American	686 (14.2)	104 (8.8)	139 (11.2)	233 (18.7)	210 (18.2)	
Other Hispanic	507 (10.5)	104 (8.8)	117 (9.4)	140 (11.3)	146 (12.6)	
Other	578 (12.0)	129 (10.9)	170 (13.7)	143 (11.5)	136 (11.8)	
Hypertension						<0.001
Yes	1825 (37.8)	379 (32.0)	434 (34.9)	491 (39.5)	521 (45.1)	
No/unknown	3004 (62.2)	806 (68.0)	811 (65.1)	752 (60.5)	635 (54.9)	
Diabetes mellitus						<0.001
Yes	574 (11.9)	81 (6.8)	124 (10.0)	177 (14.2)	192 (16.6)	
Prediabetes	4122 (85.4)	1071 (90.4)	1091 (87.6)	1035 (83.3)	925 (80.0)	
No/unknown	133 (2.8)	33 (2.8)	30 (2.4)	31 (2.5)	39 (3.4)	
Periodontitis						0.003
No	3467 (71.8)	890 (75.1)	903 (72.5)	885 (71.2)	789 (68.3)	
Yes	1362 (28.2)	295 (24.9)	342 (27.5)	358 (28.8)	367 (31.7)	
Total cholesterol	155, 202	155, 202	167, 214	173, 225	184, 235	<0.001
Triglycerides	73, 139	48, 65	80, 94	113, 137	171, 246	<0.001
HDL-C	43, 63	52, 73	47, 65	42, 59	37, 50	<0.001
LDL-C	93, 139	83, 123	94, 137	99, 144	98, 148	<0.001

Abbreviations: BMI: body mass index; RC: remnant cholesterol; HDL-C: high-density lipoprotein cholesterol; LDL-C: low-density lipoprotein cholesterol. Continuous variables were presented as mean and standard deviation, and categorical variables were expressed as *n* (%). For categorical variables, *P* values were analyzed by chi-square tests. For continuous variables, the *t*-test for slope was used in generalized linear models.

**Table 2:** Adjusted odds ratios for associations between the RC and the risk of periodontitis in NHANES 2009–2014.

Characteristic	Univariate analysis		Multivariate analysis	
	aOR (95% CI)	*P* value	aOR (95% CI)	*P* value
RC (mg/dL)		0.003		0.027
Grade 1 (<15)	Reference		Reference	
Grade 2 (15~20)	1.143 (0.953-1.370)	0.149	1.107 (0.915-1.338)	0.297
Grade 3 (20~30)	1.220 (1.019-1.461)	0.030	1.152 (0.951-1.395)	0.148
Grade 4 (>30)	1.403 (1.171-1.681)	<0.001	1.341 (1.105-1.629)	0.003

Adjusted to age, gender, BMI, marital status, race, hypertension, and diabetes mellitus. Abbreviations: BMI: body mass index; RC: remnant cholesterol; CI: confidence interval; aOR: adjusted odds ratio.

## Data Availability

The data that support the findings of this study are available from the corresponding author upon reasonable request.

## Supplementary Materials

Supplementary Materials Figure S1: the standardized mean difference (SMD) results of different variables after PSM.
