# Supplementary figures and images for: Remnant Cholesterol as an Independent Predictor of Periodontitis: A Population-Based Study
Source: Dis Markers. 2023 Feb 14;2023:3413356. doi: 10.1155/2023/3413356 (PMC9943602; doi:10.1155/2023/3413356)

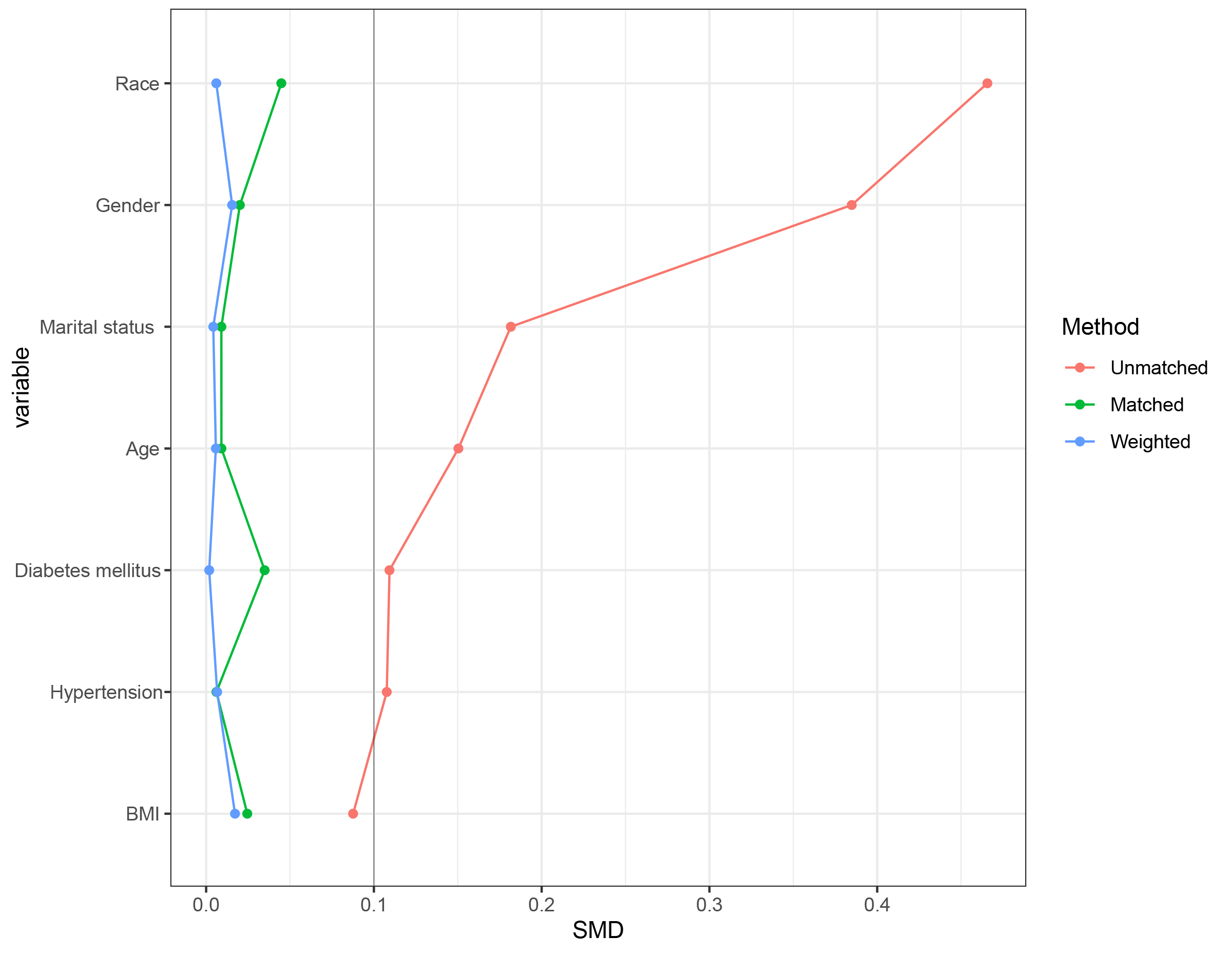

Supplement: Supplementary Materials — Figure S1: the standardized mean difference (SMD) results of different variables after PSM. [file 3413356.f1.png]
